# Impact of gestational diabetes mellitus on the duration of breastfeeding in primiparous women: an observational cohort study

**DOI:** 10.1186/s13006-021-00369-1

**Published:** 2021-02-16

**Authors:** Merja K. Laine, Hannu Kautiainen, Mika Gissler, Pirjo Pennanen, Johan G. Eriksson

**Affiliations:** 1 Department of General Practice and Primary Health Care, University of Helsinki and Helsinki University Hospital, Helsinki, Finland; 2 Folkhälsan Research Center, Helsinki, Finland; 3 Primary Health Care Unit, Kuopio University Hospital, Kuopio, Finland; 4 Information Services Department Finnish Institute for Health and Welfare (THL), Helsinki, Finland; 5 Department of Neurobiology, Care Sciences and Society, Karolinska Institute, Stockholm, Sweden; 6 Vantaa Health Center, Vantaa, Finland; 7 National University Singapore, Yong Loo Lin School of Medicine, Human Potential Translational Research programme and Department of Obstetrics and Gynecology, Singapore, Singapore; 8 Singapore Institute for Clinical Sciences (SCIS), Agency for Science, Technology and Research (A*STAR), Singapore, Singapore

**Keywords:** Breastfeeding, Educational attainment, Gestational diabetes mellitus, Obesity, Offspring, Overweight, Primiparous, Sex, Smoking, Young

## Abstract

**Background:**

The impact of gestational diabetes mellitus (GDM) on the duration of breastfeeding varies between shortening and no impact. Breastfeeding seems to reduce both maternal and offspring risk for type 2 diabetes and offspring risk for overweight or obesity later in life. The aim of our study was to evaluate in primiparous women whether GDM had an influence on the duration of breastfeeding, and further, to evaluate the factors that influenced on the duration of breastfeeding.

**Methods:**

The study cohort (*N* = 1089) consisted of all primiparous women with a Finnish background excluding women with pre-existing diabetes mellitus who lived in the city of Vantaa, Finland, gave birth to a singleton living child between 2009 and 2015, and with valid data on breastfeeding available. The diagnosis of GDM was based on a standard 75 g 2-h oral glucose tolerance test. Data were obtained from Finnish national registers and from the medical records of the city of Vantaa.

**Results:**

No differences were observed in the duration of breastfeeding between women diagnosed with GDM and without GDM, 7.5 (Standard Deviation [SD] 3.7) months versus 7.9 (SD 3.5) months (*p* = 0.17). Women diagnosed with GDM breastfed boys for a longer duration than girls (maternal age, pre-pregnancy body mass index, marital status, educational attainment, duration of pregnancy, and smoking habits adjusted *p* = 0.042). Women who breastfed < 6 months were younger, were more likely smokers, had shorter education, and higher pre-pregnancy body mass index than women who breastfed over 6 months (*p* <  0.001 for linearity).

**Conclusions:**

In primiparous women GDM did not influence breastfeeding duration. The positive health effects of breastfeeding should be emphasized especially in young, overweight and less educated women in order to minimize the risk of obesity and type 2 diabetes for themselves and their offspring.

## Background

Gestational diabetes mellitus (GDM) is a common metabolic abnormality in pregnancy and globally, the prevalence of GDM has been estimated to 13% in 2019 [1]. GDM increases the risk of short- and long-term adverse health outcomes, such as an increased risk of developing metabolic and cardiovascular disorders later in life, both for the mother and offspring [2, 3]. Breastfeeding has been reported to decrease maternal risk for ovarian cancer and type 2 diabetes and offspring risk for overweight or obesity and type 2 diabetes [4–9]. Typically, in high-income countries the duration of breastfeeding is shorter than in middle- and low-income counties [7].

Previous studies have shown inconsistent findings between GDM and the duration of breastfeeding [10]. Some studies have reported that women with GDM have a shorter duration of breastfeeding than women without GDM [11–14] whereas some studies observed no difference [5, 6, 15–17]. Lactation difficulties among women with GDM are at least partly explained by their higher likelihood of obesity and delivery complications, including cesarean section, compared women without Gestational diabetes mellitus [18, 19]. Further, neonates of women with GDM seem to have an increased risk for both low birthweight and macrosomia as well as admissions to neonatal intensive care unit, all of which may have a detrimental influence on breastfeeding [20, 21]. Most previous data originate from study cohorts consisting of both primiparous and multiparous women. Data on the influence of GDM on the duration of breastfeeding in primiparous women is missing.

The aim of the study was to evaluate whether GDM has an influence on the duration of breastfeeding, and further, to evaluate factors that influenced duration of breastfeeding in primiparous women.

## Methods

### Study population

This study is an observational cohort study from the city of Vantaa, Finland. Vantaa is the fourth most populated city in Finland with 211,000 inhabitants in 2015 in the Helsinki metropolitan area. The study cohort consisted of all Finnish primiparous women without diabetes mellitus who lived in the city of Vantaa, gave birth to a singleton living child between the 1st of January 2009 and the 31st of December 2015, and whose data on breastfeeding until the offspring age of 2 years were available (*N* = 1089). Women were defined as Finnish if they were born in Finland and their mother tongue was Finnish or Swedish.

### Maternal characteristics

Data on maternal characteristics were obtained from the Finnish Medical Birth Register maintained by the Finnish Institute for Health and Welfare. This register receives information on all live births and stillbirths from gestational weeks 22 or a birthweight of 500 g onwards from all Finnish maternity hospitals. From the Finnish Medical Birth Register we obtained data on the women’s age, status of cohabiting and smoking, pre-pregnancy weight and height, number of previous deliveries, number of fetuses, use of infertility treatments, duration of pregnancy at the day of delivery, delivery mode, and the presence of Gestational diabetes mellitus [22]. Pre-pregnancy body mass index (BMI) was calculated as pre-pregnancy weight (kg) divided by height (m) squared.

In Finland since 2008, GDM screening has been done using a 75 g 2-h oral glucose tolerance test between 24 to 28 gestation weeks in all pregnant women except those who are at low risk for GDM [23]. Nulliparous women aged less than 25 years with BMI 18.5–24.9 kg/m^2^ and without a first-degree family history of diabetes are defined as low-risk women. If a woman has one or more pathological glucose value in oral glucose tolerance test with the following diagnostic thresholds it leads to GDM diagnosis: fasting plasma glucose ≥5.3 mmol/L, 1-h glucose ≥10.0 mmol/L, and 2-h glucose ≥8.6 mmol/L [23]. Gestational diabetes mellitus screening is mainly made in public antenatal clinics in primary healthcare centers and it is free-of-charge for women. The coverage of the use of the services of public antenatal clinics is as high as 99.7–99.8% [24].

### Educational attainment, taxable incomes and chronic diseases

Data on educational attainment based on a national classification of years of schooling were obtained from Statistics Finland [25]. Data on maternal earned and capital taxable income were obtained from the Finnish Tax Administration. For annual income, each participant’s mean taxable income for three preceding years before delivery were used. The annual incomes were deflated for the year 2020 value by a consumer price index [25]. Data on women’s chronic diseases over 3 years before conception were obtained from the Social Insurance Institution [26]. In Finland, medication for certain chronic diseases is reimbursed at a rate of 65% or 100% based on a medical certificate prepared by the treating physician. The medical certificate contains the history and status observations of the person with a chronic disease. The expert physicians of the Finnish Social Insurance Institution review the certificate. When the reimbursement criteria for a chronic disease are fulfilled, the applicant receives a right to a reimbursable medication and at the same timepoint the entitlement is entered into a nationwide register.

### Breastfeeding

Data on duration of breastfeeding were obtained from the healthcare records and based on regular follow-up visits at public child welfare clinics. Breastfeeding included both exclusive, predominant, and partial breastfeeding. The Finnish national guidelines call for predominant breastfeeding for 4 to 6 months and partial breastfeeding for 1 year [27]. The visits to the public child welfare clinics are free-of-charge. In Finland, children aged 2 years or less visit the child welfare clinics at age of 1 to 4 weeks, 4 to 6 weeks, 2 months, 3 months, 4 months, 5 months, 6 months, 8 months, 10 months, 12 months, 18 months, and 24 months [28]. The coverage of the public child welfare clinic use is as high as 99.6% [28].

### Offspring birth characteristics

Data on offspring sex, birthweight, Apgar score at 1 min, and admissions to neonatal intensive care unit or need for respirator treatment before the age of 7 days were obtained from the Finnish Medical Birth Register.

### Data combination

In Finland, every citizen and permanent resident has a personal identification number. With the personal identification number register data from the Finnish Medical Birth Register, Statistics Finland, the Finnish Tax Administration, the Social Insurance Institution, and the healthcare records from the child welfare clinics were combined at an individual level.

### Statistical analyses

Data are presented as means with SD or as counts (*n*) with percentages (%). Statistical comparisons between the GDM groups were made using the t-test, analysis of variance (ANOVA), chi-square test, or Fisher-Freeman test. The hypothesis of linearity across duration of breastfeeding (< 6 months, 6–11 months and ≥ 12 months) were evaluated by using the Cochran-Armitage test for trend and analysis of variance with an appropriate contrast (orthogonal polynomial). The Kaplan-Meier method was applied to estimate the cumulative probability of breastfeeding women diagnosed with and without Gestational diabetes mellitus. We used Cox proportional hazards model to calculate the adjusted hazard ratios (HR). The normality of variables was evaluated graphically and using Shapiro–Wilk W test. Stata 16.1 (StataCorp LP; College Station, Texas, USA) statistical package was used for the analysis.

## Results

Characteristics of the primiparous women and their offspring by presence of GDM are shown in the Table 1. Women diagnosed with GDM were older (30.1 [SD 4.8] years versus 28.7 [SD 4.8] years, *p* <  0.001) and had higher pre-pregnancy BMI (26.9 [SD 5.4] kg/m^2^ versus 23.3 [SD 3.7] kg/m^2^, *p* <  0.001) than women in pregnancies without GDM (Table 1). No differences were observed in marital status, educational attainment, annual maternal incomes or smoking habits (Table 1). Further, no differences were observed in the offspring characteristics assessed (Table 1).

**Table 1:** Characteristics of primiparous women (*N* = 1089) and their offspring by the presence of gestational diabetes mellitus

	Women without GDM (***n*** = 934)	Women with GDM (***n*** = 155)	***P*** - value
**Maternal characteristics**			
Age (years), mean (SD)	28.7 (4.8)	30.1 (4.8)	< 0.001
Married, n (%)	469 (50)	81 (52)	0.64
Smokers			0.38
Non-smokers, n (%)	803 (86)	128 (83)	
Quitted during the first trimester, n (%)	69 (7)	12 (8)	
Smokers over pregnancy, n (%)	62 (7)	15 (10)	
Education years, mean (SD)	13.8 (2.5)	13.8 (2.5)	0.93
Annual maternal income (€), mean (SD)	26,280 (13,160)	27,064 (13,768)	0.50
Pre-pregnancy body mass index (kg/m^2^), mean (SD)	23.3 (3.7)	26.9 (5.4)	< 0.001
Pregnancies without fertility treatments, n (%)	842 (90)	141 (91)	0.75
Duration of pregnancy (weeks), mean (SD)	40.1 (1.5)	39.6 (2.1)	< 0.001
Vaginal deliveries, n (%)	749 (80)	120 (77)	0.43
Chronic diseases			
Lung diseases, n (%)	21 (2)	6 (4)	0.23
Rheumatoid diseases, n (%)	9 (1)	0 (0)	0.37
Mental diseases, n (%)	7 (1)	1 (1)	0.98
Thyroid diseases, n (%)	2 (0)	0 (0)	0.99
**Infant characteristics**			
Sex (boy), n (%)	471 (50)	88 (57)	0.14
Birthweight (grams), mean (SD)			
Girls	3374 (467)	3403 (651)	0.65
Boys	3533 (478)	3516 (555)	0.76
Apgar score at one minute, mean (SD)	8.5 (1.3)	8.5 (1.4)	0.93
Admission to neonatal intensive care unit, n (%)	76 (8)	15 (10)	0.52
Need for respirator treatment, n (%)	17 (2)	2 (1)	0.98

*GDM* gestational diabetes mellitus, *SD* standard deviation

**Fig. 1:**
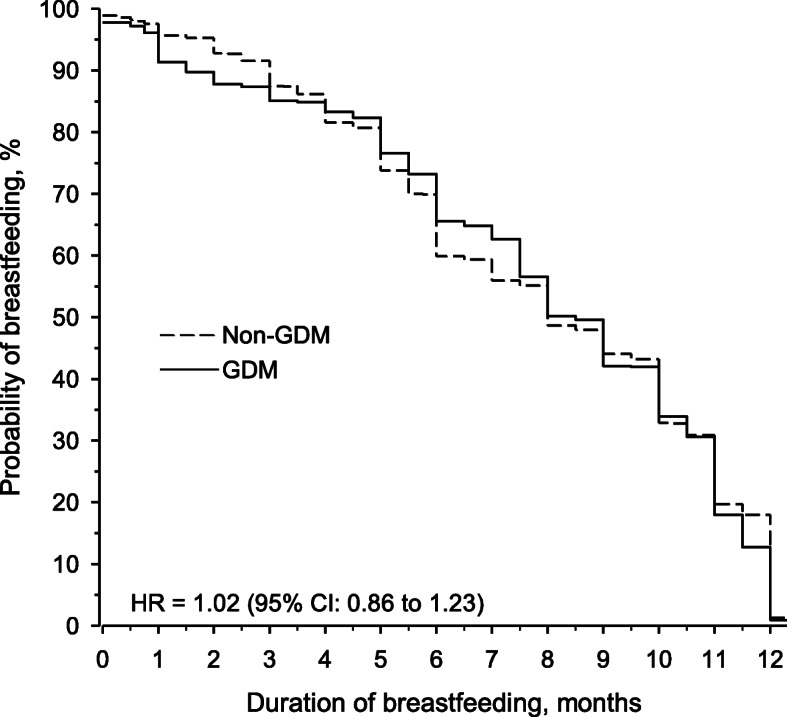
Probability of breastfeeding in primiparous women diagnosed with and without gestational diabetes mellitus. Hazard ratio is adjusted for maternal age, pre-pregnancy body mass index, marital status, educational attainment, duration of pregnancy, smoking habits, and infant’s sex. GDM = gestational diabetes mellitus; HR = hazard ratio

Figure 1 shows the probability of any breastfeeding after adjusting for maternal age, pre-pregnancy BMI, marital status, educational attainment, duration of pregnancy, smoking habits, and infant’s sex in women with and without Gestational diabetes mellitus. Mean duration of breastfeeding was 7.5 (SD 3.7) months in women diagnosed with GDM and 7.9 (SD 3.5) months in women without GDM (*p* = 0.17). Of the women diagnosed with GDM, five (3%) did not initiate breastfeeding at all and the corresponding number was nine (1%) among the women without Gestational diabetes mellitus (*p* = 0.037).

**Fig. 2:**
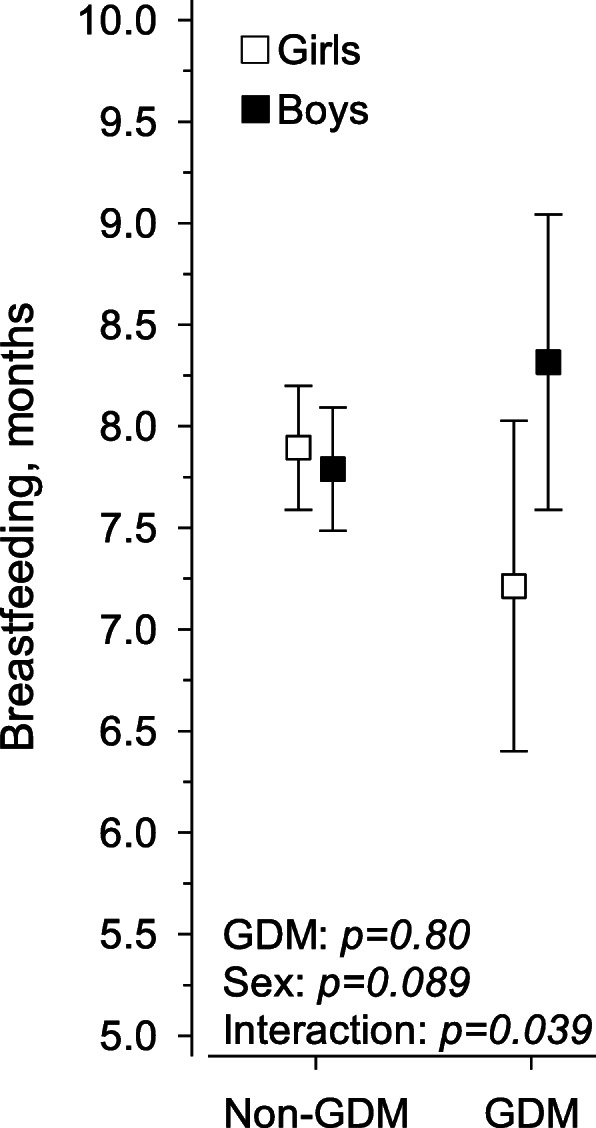
Impact of offspring sex and gestational diabetes mellitus and their interaction on the duration of breastfeeding in women diagnosed with and without gestational diabetes mellitus, adjusted for maternal age, pre-pregnancy body mass index, marital status, educational attainment, duration of pregnancy, and smoking habits. GDM = gestational diabetes mellitus

Women diagnosed with GDM breastfed boys for a longer time than girls (maternal age, pre-pregnancy BMI, marital status, educational attainment, duration of pregnancy, and smoking habits adjusted *p* = 0.042) (Fig. 2).

Table 2 shows the characteristics of primiparous women and their offspring by the duration of breastfeeding. Women who breastfed < 6 months were younger, more likely smokers, had lower educational attainment, and had higher pre-pregnancy BMI than women who breastfed 6–11 months or ≥ 12 months (for all *p* <  0.001 for linearity) (Table 2). Further, women who breastfed < 6 months were more often unmarried and had lower annual income than women who breastfed 6–11 months or ≥ 12 months (for all *p* = 0.002 for linearity) (Table 2). No differences were observed in the characteristics of the offspring (Table 2).

**Table 2:** Characteristics of primiparous women (*N* = 1089) and their offspring by the duration of breastfeeding

	< 6 months ***n*** = 329	6–11 months ***n*** = 569	≥ 12 months ***n =*** 191	***p*** for linearity
**Maternal characteristics**				
Age (years), mean (SD)	28.2 (5.6)	29.0 (4.4)	29.7 (4.4)	< 0.001
Married, n (%)	140 (43)	305 (54)	105 (55)	0.002
Smokers				< 0.001
Non-smokers, n (%)	262 (80)	498 (88)	171 (90)	
Quitted during the first trimester, n (%)	22 (7)	44 (8)	15 (8)	
Smokers over pregnancy, n (%)	45 (14)	27 (5)	5 (3)	
Education years, mean (SD)	13.0 (2.5)	14.0 (2.4)	14.5 (2.4)	< 0.001
Annual maternal income (€), mean (SD)	24,500 (14,100)	27,000 (12,900)	28,000 (12,400)	0.002
Pre-pregnancy body mass index (kg/m^2^), mean (SD)	24.6 (4.8)	23.5 (3.9)	23.2 (3.7)	< 0.001
Pregnancies without fertility treatments, n (%)	303 (92)	510 (90)	170 (89)	0.20
Duration of pregnancy (weeks), mean (SD)	39.8 (1.8)	40.2 (1.4)	40.0 (1.7)	0.060
Vaginal deliveries, n (%)	258 (78)	455 (80)	156 (82)	0.37
Gestational diabetes mellitus, n (%)	54 (16)	80 (14)	21 (11)	0.088
Chronic diseases				
Lung diseases, n (%)	6 (2)	15 (3)	6 (3)	0.33
Rheumatoid diseases, n (%)	3 (1)	4 (1)	2 (1)	0.94
Mental diseases, n (%)	4 (1)	3 (1)	1 (1)	0.30
Thyroid diseases, n (%)	0 (0)	2 (0)	0 (0)	0.79
**Infant characteristics**				
Sex (boy), n (%)	166 (50)	292 (51)	101 (53)	0.60
Birthweight (grams), mean (SD)				
Girls	3373 (577)	3390 (448)	3348 (467)	0.81
Boys	3527 (520)	3529 (467)	3540 (514)	0.85
Apgar score at 1 min, mean (SD)	8.5 (1.4)	8.5 (1.3)	8.5 (1.2)	0.61
Admission to neonatal intensive care unit, n (%)	35 (11)	39 (7)	17 (9)	0.30
Need for respirator treatment, n (%)	11 (3)	5 (1)	3 (2)	0.057

## Discussion

We observed no differences in the duration of breastfeeding between primiparous women diagnosed with GDM and those without Gestational diabetes mellitus. The women breastfed their offspring for an average of almost 8 months. Women with GDM breastfed boys for a longer time than girls. Women who breastfed their offspring for a long time were typically older, slimmer, non-smokers, and better educated than women who breastfed for a short time.

Our study findings endorse previous study findings that GDM does not influence duration of breastfeeding [5, 6, 16]. In this cohort including Finnish primiparous women, the average duration of breastfeeding was almost 8 months. Further, 70% of the women breastfed their offspring for at least 6 months and almost 20% at least 12 months. Previous studies have reported that in high income countries around 45% of women breastfed their offspring for at least 6 months and around 25% for at least 12 months [7]. The variations are considerable, for example in Norway around 70% of women breastfed their offspring for at least 6 months and around 35% at least 12 months, and in Denmark around 15% at least 6 months and 3% at least 12 months, respectively [7]. At least in part, the large differences in breastfeeding duration are explained by the fact that the duration of maternity leave varies greatly from country to country. In Finland, mothers have an entitlement for 105 working days maternity leave paid by the Finnish Social Insurance Institution [29]. In addition, after the maternity leave the Finnish Social Insurance Institution pays parental allowance, either to mother or father, for 158 working days [30]. All in all, this means that a child is typically the first 9 months at homecare. After the parental allowance, if the child is taken care of at home, the parents are entitled to a child home allowance paid by the Finnish Social Insurance Institution until the child is 3 years old [31]. Further during the past 10 years in Finland, the counselling for breastfeeding has been intensified both in maternity hospitals and maternity clinics [32].

Interestingly, we observed that women diagnosed with GDM breastfed their boys longer than girls. To the best of our knowledge, no previous studies have report findings on the presence of Gestational diabetes mellitus, offspring’s sex and duration of breastfeeding. Overall, previous study findings on the association between offspring’s sex and duration of breastfeeding have been inconsistent [21, 33–36]. In the Newcastle Thousand Families study no sex difference in the duration of breastfeeding was reported [36]. Similarly, in a Chinese study no sex differences in relation to duration of breastfeeding were found [21]. Contrary in an Indian study, girls were breastfed for shorter periods than boys [35]. In a US study no gender differences were observed in the duration of breastfeeding except in Hispanic mothers who breastfed their boys for shorter time than girls [34]. Breastfeeding related issues are obviously highly related to cultural factors [17, 34]. However, the underlying factors explaining our study observations that women diagnosed with GDM breastfed their boys longer than girls remains unclear.

According to our observations, primiparous women who breastfed for a long time were characterized by higher age than women who breastfed a short time. This observation is line with previous study including both primiparous and multiparous women [37]. Further, endorsing previous studies we found that women with higher degree of adiposity, breastfed their offspring for shorter duration than slimmer women [12, 37–40]. There are some evidence that overweight and obese women may have increased progesterone concentrations and/or decreased prolactin response to infant suckling leading breastfeeding problems [18]. Also, large breasts may make it more challenging for the infant to achieve a correct latch [18]. In line with previous studies, we found that non-smokers, higher educated, and married women had a longer duration of breastfeeding [20, 37, 39]. Some studies have shown that delivery complications, such as Caesarean section, or serious health problems of the newborn lead to lactation difficulties [19, 20, 38]. We did not observe such an influence. A long duration of breastfeeding seems to associate with beneficial health outcomes for the mother and offspring [4–9].

Our study has several strengths. Data on deliveries, maternal characteristics, and infant’s birth characteristics were based on the Finnish Medical Birth Register, which has been found to be of good quality [41]. Data on educational attainment, taxable incomes, and chronic diseases were based on reliable register data. Breastfeeding data were documented in the medical records by healthcare professionals. We studied only primiparous women to avoid biases of previous pregnancies and breastfeeding.

### Study limitations

We had only data on any breastfeeding, not separately on exclusively, predominant and partial breastfeeding. We were missing on data on women’s dietary and physical activity habits as well as gestational weight gain, which all may influence the duration of breastfeeding. In our study cohort, the number of women who did not initiate breastfeeding at all was low; this study finding would be needed to confirm in a larger study population. Further, all women were Finnish, thus, the generalizability of our study observations is limited.

## Conclusions

In a high-income country like Finland with a long maternity leave and a well-functioning public free-of-charge antenatal and children welfare clinic organization, GDM had no influence on the duration of breastfeeding. The positive health effects of breastfeeding should be emphasized especially in young, overweight and less educated women in order to minimize the risk of obesity and type 2 diabetes for themselves and their offspring.

## Data Availability

Data cannot be shared for both legal and ethical reasons. Data from the Finnish Institute for Health and Welfare (THL), Statistics Finland, the Finnish Social Insurance Institution, and the city of Vantaa can only be used for the purpose stated in the license granted, scientific research on society by the license applicant, and can therefore not be shared with third parties.

## Abbreviations

BMI: body mass index
CI: confidential intervals
GDM: gestational diabetes mellitus
HR: hazard rate
SD: standard deviation
